# Association between skeletal morphology and agenesis of all four third molars in Japanese orthodontic patients

**DOI:** 10.1007/s10266-017-0336-z

**Published:** 2018-01-12

**Authors:** Yoshiko Sugiki, Yoshiki Kobayashi, Miwa Uozu, Toshiya Endo

**Affiliations:** 1 Orthodontic Dentistry, The Nippon Dental University Niigata Hospital, 1-8 Hamaura-cho, Chuo-ku, Niigata, 951-8580 Japan; 2 Orthodontics and Dentofacial Orthopedics, Field of Oral and Maxillofacial Growth and Development, Course of Clinical Science, The Nippon Dental University Graduate School of Life Dentistry at Niigata, 1-8 Hamaura-cho, Chuo-ku, Niigata, 951-8580 Japan; 3 Department of Orthodontics, The Nippon Dental University School of Life Dentistry at Niigata, 1-8 Hamaura-cho, Chuo-ku, Niigata, 951-8580 Japan

**Keywords:** Skeletal morphology, Third molar agenesis, Cephalogram, Japanese orthodontic patients, Facial pattern

## Abstract

The purpose of this study was to clarify differences in skeletal morphologies between male and female orthodontic patients with and without agenesis of all four third molars. A total of 64 patients (32 males and 32 females) with agenesis of all four third molars without agenesis of other teeth were selected as the third molars agenesis group (group 1). In addition, 64 patients (32 males and 32 females) with all these teeth were selected as controls (group 2). Lateral cephalograms taken between the ages of 14 and 30 years were used to compare skeletal morphology between groups 1 and 2 and between sexes. Maxillary length (*P* < 0.001), lower facial height (*P* < 0.05), gonial angle (*P* < 0.001) and mandibular plane angle (*P* < 0.001) were significantly smaller in group 1 than in group 2. Irrespective of the presence or absence of all four third molars, males had significantly smaller lower facial height (*P* < 0.01) and mandibular plane angle (*P* < 0.001) and significantly greater total mandibular length (*P* < 0.001), mandibular body length (*P* < 0.001) and mandibular ramus height (*P* < 0.001) than females. Japanese orthodontic patients with agenesis of all four third molars had significantly small maxillary length, lower facial height, gonial angle and mandibular plane angle.

## Introduction

Agenesis of third molars (M3s) occurs most frequently in orthodontic populations [1, 2]. An abundance of the literature has reported a wide range in the prevalence rate of M3 agenesis in different populations [3–5], and most studies have indicated no significant differences in prevalence rates according to sex [2, 3, 5]. In Japanese populations, the prevalence of M3 agenesis has ranged from 22.2 [6] to 32.3% [2]. A recent study showed that the prevalence of subjects with of agenesis of all four M3s and without agenesis of other teeth was 4% of Japanese orthodontic patients [2].

Several studies have indicated significant associations between M3 agenesis and agenesis of other teeth, including maxillary lateral incisors and maxillary and mandibular second premolars [1, 2, 7–11]. Additionally, orthodontic subjects with M3 agenesis exhibit short maxillary length [12, 13] and mandibular length [14, 15], small gonial angle [16] and mandibular plane angle [14, 16, 17] and reduced lower facial height [14, 17]. Some studies have showed that orthodontic patients with skeletal Class II malocclusion have significantly lower prevalence of agenesis of M3s [6] and other teeth than M3s [18, 19] than those with skeletal Class III malocclusion, thus suggesting that skeletal pattern may have influence on tooth agenesis. In most of these studies on the associations of craniofacial morphology and agenesis of M3s, orthodontic patients were divided into groups according to the number or location of congenitally missing M3s [12, 13, 16, 17]. Woodworth et al. [14] and Tavajohi-Kermani et al. [15] selected orthodontic subjects with tooth agenesis without restriction to M3s. These studies did not assess the influence of sex differences on craniofacial morphology of patients with M3 agenesis [12–17].

To our knowledge, no studies have assessed in detail the skeletal morphology in orthodontic patients with agenesis of all this molar and without agenesis of other teeth. Therefore, the purpose of this study was to clarify skeletal morphologies in male and female orthodontic patients with agenesis of all four M3s and without agenesis of other teeth, relative to those patients with all these teeth.

## Materials and methods

This study was approved by the Research Ethics Committee of our institution (Approval No. ECNG-R-306).

### Sample

A total of 436 Japanese patients (163 males and 273 females) with and without M3 agenesis were retrospectively selected from orthodontic files of patients that had visited our institution between January 1994 and December 2015. Informed consent was obtained from all individual participants included in the study. The criteria for inclusion in this study were patients with availability of panoramic radiographs and lateral cephalograms taken between the ages of 14 and 30 years in the same day and with full eruption of all maxillary and mandibular teeth up to the second molars. The criteria for exclusion were patients with agenesis of other teeth than M3s, with congenital deformities, such as craniofacial syndrome or clefts, with history of extraction of a permanent tooth, and with history of orthodontic treatment prior to visiting our hospital. Patients with a Class I skeletal base had no lateral cephalograms taken when the orthopantomogram was taken if the orthodontist did not require it. These patients were immediately excluded from this study.

Among the 436 patients, 228 (102 males and 126 females) had agenesis of one or more M3s. The remaining 208 patients (61 males and 147 females) had all four M3s. A study subject of 74 patients (36 males and 38 females) without all four M3s was selected from 228 patients with agenesis of one or more M3s. One hundred fifty-four patients were excluded from the 228 patients initially selected from orthodontic patient files because they had M3 agenesis that did not fit the agenesis patterns in this study. Thirty-two males with a mean age of 18 years and 6 months (SD, 3 years and 4 months; range, 14 years 2 months–25 years 7 months) and 32 females with a mean age of 19 years and 1 month (SD, 3 years and 4 months; range, 14 years–25 years 9 months) were randomly selected from 74 patients without all four M3s as the M3 agenesis group (group 1). As the control group (group 2), 32 males with a mean age of 17 years and 10 months (SD, 2 years and 4 months; range, 14 years 4 months–23 years 2 months) and 32 females with a mean age of 17 years and 10 months (SD, 2 years and 4 months; range, 14 years 3 months–21 years 9 months) were randomly chosen from 208 patients with four M3s. To make the random selection, patients studied were coded and then selected in each group by a person who was not directly involved in this study.

Using G* Power version 3 (Heinrich Heine University, Dusseldorf, Germany), a post hoc power analysis was performed to determine the power of two-way analysis of variance (ANOVA) at an effect size of 0.25 (Cohen’s medium effect size), alpha error probability of 0.05, degree of freedom of 1, number of groups of 4 and a sample size of 128 [20]. The power was 0.80, thus demonstrating that the sample size in each group was sufficient for statistical comparisons.

### Tooth agenesis

Panoramic radiographs were mainly used to examine tooth agenesis. All panoramic radiographs were taken with the same system (Veraview epocs X550, Morita Corporation, Kyoto, Japan). Tooth agenesis was diagnosed when there was no sign of crown mineralization on panoramic radiographs and no history of extraction of this tooth. A tooth was defined as not missing when more than 3/4 of its crown appeared to be mineralized on panoramic radiographs. If necessary, medical and dental records were examined to confirm any history of tooth extraction.

### Cephalometric analysis

**Fig. 1:**
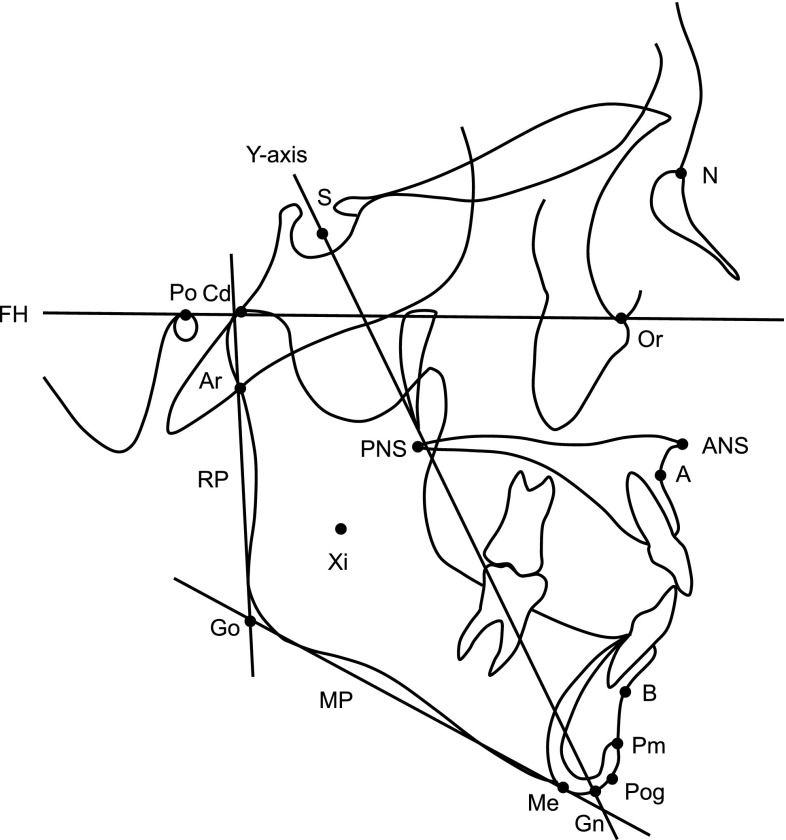
Reference points and lines used. *S* sella turcica, *N* nasion, *Or* orbitale, *Po* porion, *Ar* articulare, *ANS* anterior nasal spine, *PNS* posterior nasal spine, *A* point A, *B* point B, *Pog* pogonion, *Gn* gnathion, *Me* menton, *Go* gonion, *Cd* condylion, *Xi* Xi point, *Pm* protuberance menti, *FH* Frankfort horizontal plane, *PP* palatal plane, *MP* mandibular plane, *RP* ramus plane

All cephalograms were taken using the same cephalostat (CX-150SK, Asahi roentgen, Kyoto, Japan) and with standardized settings. Cephalograms of each patient were coded by a person who was not directly involved in this study, and each was traced and measured. Sixteen reference points and four reference lines were selected, and four linear and eight angular measurements were taken to investigate the association between maxillofacial morphology and M3 agenesis (Fig. 1). Angular and linear measurements were made to the nearest 0.1° and 0.1 mm, respectively, with the aid of a computer system containing the WinCeph analysis software program (Rise Corp, Miyagi, Japan).

To avoid any measurement bias, one investigator (YS), who was blinded to the groups, measured the coded cephalograms. The results of all measurements were subsequently sorted by group and sex for statistical comparison.

### Statistical analysis

Statistical analyses were performed using a commercially available statistical package SPSS version 17.0 J (SPSS Japan Inc., Tokyo, Japan). Means and standard deviations (SDs) were calculated for each measurement within each group and each sex. Two-way ANOVA was performed to test the main effects of sex and group. *P* values < 0.05 were considered statistically significant.

### Measurement error

To assess measurement errors, forty coded cephalograms were randomly selected and re-examined by the same investigator (YS) for a second time 3 months later. Student’s *t* test with a 95% confidence interval did not reveal any systematic errors. Random errors, calculated according to the Dahlberg formula [21], did not exceed ± 0.49° or ± 0.44 mm, which were unlikely to spoil the significant results in this study.

## Results

Two-way ANOVA showed no significant differences in mean ages between the two groups or between sexes and no significant interactions between the two variables.

Table 1 shows the means and SDs for all measurements in the two groups.

**Table 1:** Results of liner and angular measurements

Measurement	Sex	Group 1 (*n* = 64)		Group 2 (*n* = 64)	
		Mean	SD	Mean	SD
ANS–PNS (mm)	Male	51.96	2.31	53.86	2.81
	Female	49.23	2.08	52.43	3.13
Cd–Gn (mm)	Male	125.41	6.80	126.00	8.66
	Female	120.0	5.24	118.90	7.62
Go–Pog (mm)	Male	82.12	4.49	82.0	5.22
	Female	78.91	4.73	77.71	5.73
Co–Go (mm)	Male	61.37	6.68	61.57	5.12
	Female	57.47	5.23	58.70	5.27
SNA (°)	Male	82.15	4.15	82.67	2.87
	Female	81.23	3.45	81.66	3.23
ANS–Xi–PM (°)	Male	47.24	3.35	49.49	3.47
	Female	49.19	2.49	50.62	3.93
ANB (°)	Male	2.37	2.29	2.45	3.06
	Female	2.60	2.97	3.94	3.02
MP–FH (°)	Male	26.77	3.12	30.12	2.79
	Female	29.39	4.03	30.24	4.52
RP–FH (°)	Male	85.65	5.41	84.81	4.19
	Female	85.86	5.12	87.39	4.38
MP–RP (°)	Male	122.58	4.63	127.06	4.11
	Female	123.41	5.12	125.18	5.17
SNB (°)	Male	80.06	3.86	80.08	4.18
	Female	78.67	4.11	78.38	4.13
*Y*-axis (°)	Male	80.12	3.77	80.08	4.18
	Female	78.60	4.17	77.42	4.75

As shown in Tables 2 and 3, two-way ANOVA demonstrated significant differences in the ANS–PNS dimension (maxillary length, *P* < 0.001, *P* < 0.001), the ANS–Xi–Pm angle (lower facial height, *P* < 0.05, *P* < 0.01) and the MP–FH angle (mandibular plane angle, *P* < 0.001, *P* < 0.001) between groups and between sexes, respectively, with no significant interactions between the two variables. Additionally, significant differences in the Cd–Gn dimension (total mandibular length, *P* < 0.001), the Go–Pog dimension (mandibular body length, *P* < 0.001) and the Cd–Go dimension (mandibular ramus height, *P* < 0.001) were observed between sexes (Table 2). A significant difference was also observed in the MP–RP angle (gonial angle, *P* < 0.001) between groups (Table 3). However, no significant differences were observed in the SNA angle (prognathism of maxillary alveolar bone), the SNB angle (mandibular alveolar bone), the RP–FH angle (ramus inclination), the ANB angle (sagittal jaw relationship angle) or the *Y*-axis angle (*Y*-axis) between groups or between sexes (Table 3).

**Table 2:** Results of two-way ANOVA test for linear measurements

Measurement	Source	Sum of squares	Degree of freedom	Mean square	*F* value	*P* value	Comparison
ANS–PNS (mm)	Groups	167.079	1	167.079	31.374	0.000***	2 > 1
	Sexes	174.970	1	174.970	32.855	0.000***	Male > female
	Interaction	4.909	1	4.909	0.922	0.339	NS
	Error	671.006	126	5.325			
Cd–Gn (mm)	Groups	133.334	1	133.334	2.704	0.103	NS
	Sexes	1740.697	1	1740.697	35.300	0.000***	Male > female
	Interaction	124.031	1	124.031	2.515	0.115	NS
	Error	6231.200	126	49.311			
Go–Pog (mm)	Groups	19.522	1	19.552	0.734	0.393	NS
	Sexes	619.989	1	619.989	23.270	0.000***	Male > female
	Interaction	45.188	1	45.188	1.696	0.195	NS
	Error	3357.114	126	26.644			
Cd–Go (mm)	Groups	78.459	1	78.459	2.828	0.095	NS
	Sexes	760.240	1	760.240	27.405	0.000***	Male > female
	Interaction	30.368	1	30.368	1.095	0.297	NS
	Error	3495.363	126	27.741			

*ANOVA* analysis of variance, *NS* not significant

*** *P* < 0.001

**Table 3:** Results of two-way ANOVA test for angular measurements

Measurement	Source	Sum of squares	Degree of freedom	Mean square	*F* value	*P* value	Comparison
SNA (°)	Groups	5.656	1	5.656	0.483	0.488	NS
	Sexes	10.873	1	10.873	0.929	0.337	NS
	Interaction	3.708	1	3.708	0.317	0.574	NS
	Error	1474.540	126	11.703			
ANS–Xi–Pm (°)	Groups	54.776	1	54.776	4.204	0.042*	2 > 1
	Sexes	93.389	1	93.389	7.168	0.008**	Female > male
	Interaction	13.313	1	13.313	1.022	0.314	NS
	Error	1641.556	126	13.028			
ANB (°)	Groups	25.068	1	25.063	3.654	0.456	NS
	Sexes	28.376	1	28.376	4.137	0.314	NS
	Interaction	16.512	1	16.512	2.407	0.543	NS
	Error	864.320	126	6.860			
MP–FH (°)	Groups	248.868	1	248.868	15.267	0.000***	2 > 1
	Sexes	174.036	1	174.036	10.676	0.001***	Female > male
	Interaction	2.777	1	2.777	0.170	0.681	NS
	Error	2053.984	126	16.301			
RP–FH (°)	Groups	1.590	1	1.590	0.063	0.802	NS
	Sexes	14.347	1	14.347	0.569	0.452	NS
	Interaction	6.759	1	6.759	0.268	0.605	NS
	Error	3174.479	126	25.194			
MP–RP (°)	Groups	344.969	1	344.969	11.829	0.001***	2 > 1
	Sexes	48.676	1	48.676	1.669	0.199	NS
	Interaction	5.379	1	5.379	0.184	0.668	NS
	Error	3674.494	126	29.163			
SNB (°)	Groups	0.162	1	0.162	0.010	0.922	NS
	Sexes	61.125	1	61.125	3.656	0.058	NS
	Interaction	0.002	1	0.002	0.000	0.991	NS
	Error	2106.807	126	16.721			
*Y*-axis (°)	Groups	1.032	1	1.032	0.092	0.762	NS
	Sexes	22.067	1	22.067	1.970	0.163	NS
	Interaction	0.170	1	0.170	0.015	0.902	NS
	Error	1411.243	126	11.200			

*ANOVA* analysis of variance, *NS* not significant

* *P* < 0.05; ** *P* < 0.01; *** *P* < 0.001

## Discussion

The panoramic radiographs taken between the ages of 14 and 30 years were used for diagnosis of tooth agenesis in this study. This minimum age of 14 years was based on the suggestion of Garn and Lewis [7] that the upper age limit for M3 agenesis is 14 years. Some studies have adopted a maximum age of 21 years [2, 22, 23]. The adaptation is based on the fact that eruption of M3s begins at 14–21 years of age [21]. However, Rakhshan [24] reported that there were no limits over the maximum age as long as positive extraction history is an exclusion criterion. Therefore, the maximum age of subjects in this study was 30 years.

Previous studies indicated that there were significant correlation coefficients between skeletal and dental maturity stages and that the second molars showed the highest correlation and the M3s showed the lowest relationship for both males and females [25, 26]. In this study, subjects with full eruption of all maxillary and mandibular teeth up to the second molars were selected and there were no significant differences in the mean ages between groups 1 and 2 or between sexes, as confirmed by two-way ANOVA. Therefore, subjects selected in this study were suitable for measuring skeletal morphology.

In this study, maxillary length, lower facial height, gonial angle and mandibular plane angle were significantly smaller in subjects without all four M3s (group 1, agenesis group) than in those with all four M3s (group 2, control group). These results were supported by previous studies that demonstrated the M3 agenesis group showed shorter maxillary length [12, 13] and decreased gonial angle [16], mandibular plane angle [16, 17] and lower facial height [17] than the control group. There were some discrepancies in sample selection between their agenesis groups and ours. Altan et al. [13] categorized their subjects with M3 agenesis into three groups: the bilateral maxillary M3 agenesis group, the bilateral mandibular M3 agenesis group and the all four M3 agenesis. Sanchez et al. [17] divided their subjects with M3 agenesis into two groups: the bilateral maxillary M3 agenesis group and the bilateral mandibular M3 agenesis group. Kajii et al. [12] also classified their subjects into two maxillary and mandibular agenesis groups, irrespective of unilateral or bilateral M3 agenesis. Moreover, Ramiro-Verdugo et al. [16] selected subjects with agenesis of at least one M3. Woodworth et al. [14] and Tavajohi-Kermani et al. [15] verified short maxillary length in subjects with agenesis of maxillary M3s and other teeth, as confirmed by the results of the present study.

Reduced maxillary length was considered to be due to inadequate apposition of bone to the tuberosity area in our M3 agenesis group. This consideration is based on a report that increased maxillary length was accomplished almost completely by apposition of bone to the maxillary tuberosity, which was associated with tooth eruption [27]. Decreased gonial angle, mandibular plane angle and lower facial height in the M3 agenesis group were presumably related to upward rotation of the mandible as a result of reduced vertical dimension of the alveolar process. This upward rotation of the mandible in the M3 agenesis group may be due to the fact that the vertical growth at the condyle was superior to the sum of the vertical growth components at facial sutures and alveolar processes in the subjects with agenesis of posterior teeth [28].

Our results demonstrated that there were no significant differences in the total mandibular length, mandibular body length, or mandibular ramus height between the subjects with and without agenesis of all four M3s. These results were consistent with the findings by Ades et al. [29] and Kaplan [30], who reported that mandibular growth pattern was no significantly different between the subject with and without M3, thus indicating that agenesis of all four M3s might not be associated with the mandibular length. Conversely, Altan et al. [13] reported that the total mandibular length was significantly smaller in patients with agenesis of all for M3s than in those with all four M3s.

From a genetic point of view, Msx1 over-expression during bone development affected craniofacial morphology and a deficiency of this expression resulted in a switch from a dolichofacial pattern to a mesiofacial pattern or brachyfacial pattern [31, 32]. Moreover, Pax9 deficiency was responsible for skeletal deficiency and agenesis of M3s [32, 33]. These pieces of evidence of Msx1 and PAX9 were in accord with our findings that skeletal morphological deviations occurred in the agenesis group.

Our results showed that irrespective of the presence or absence of all four M3s, lower facial height and mandibular plane angle were significantly smaller in males than in females, demonstrating that males had a greater tendency toward a brachyfacial pattern than females. Males have thicker masseter muscle and stronger occlusal force than females [34, 35], and subjects with strong occlusal force presented with a brachyfacial pattern [36, 37], as observed in our male subjects. Therefore, differences in the tested measurements between sexes observed in this study may be caused by the discrepancy in occlusal force generated by the masseter muscle. Conversely, Wu et al. [38] and Gu et al. [39] observed that males had larger lower facial height and increased mandibular plane angle compared with females.

In this study, total mandibular length, mandibular body length and mandibular ramus height were significantly shorter in females than in males, irrespective of the presence or absence of all four M3s. These results were consistent with findings of Gu et al. [39] and Daraze et al. [40] who reported that these mandibular dimensions were significantly shorter in females than in males. Growth at the condylar cartilage thrusts the mandible forward and downward, thus resulting in an increase in total mandibular length. Moreover, growth of the mandible by resorption along the anterior edge of the ramus and apposition along its posterior edge increases mandibular body length and mandibular ramus height [41]. Moreover, Franchi et al. [42] showed that both males and females experienced pubertal growth spurts in linear dimensions of the mandible. Mellion et al. [43] described that skeletal growth changes in the face of females slow and cease shortly after puberty; however, dimensional changes in males continue through the late adolescent period. Accordingly, sex differences in mandibular dimensions observed in this study were thought to be due to discrepancies in the mechanism of mandibular growth between sexes.

We can summarize our conclusions as follows:

- Maxillary length, lower facial height, gonial angle and mandibular plane angle were significantly smaller in orthodontic subjects without all four M3s and with other teeth than in those with all these teeth.
- Irrespective of the presence or absence of all four M3s, males had significantly smaller lower facial height and mandibular plane angle and significantly greater total mandibular length, mandibular body length and mandibular ramus height than females.
